# Supplementary material for: Identification of photoperiod-induced specific miRNAs in the adrenal glands of Sunite sheep (Ovis aries)
Source: Front Vet Sci. 2022 Jul 22;9:888207. doi: 10.3389/fvets.2022.888207 (PMC9354845; doi:10.3389/fvets.2022.888207)
Supplement: Supplementary file 1 [file Data_Sheet_1.ZIP › Supplementary file/miRDeep2 file.zip!/miRDeep2 file/Know_Structure/LP42A2/oar-mir-103.pdf]

miRBase precursor	: oar-mir-103
Total read count	: 14773
oar-miR-103 read count	: 14772
remaining reads	: 1

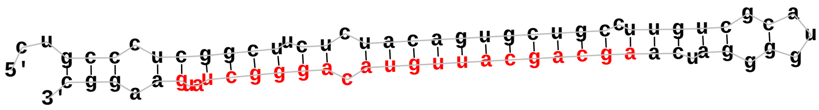

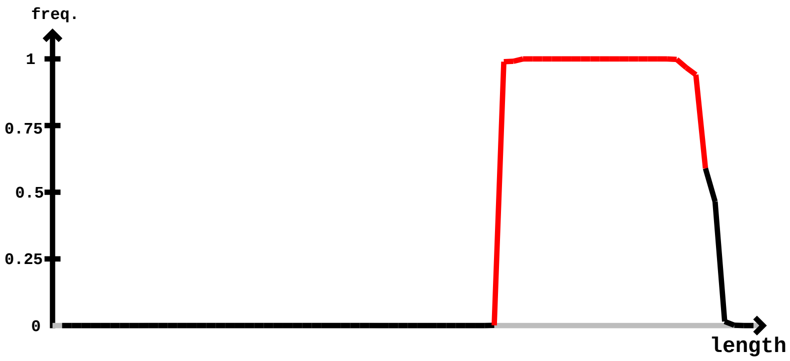


```
                                             oar-miR-103
5'- cugcccucggcuucucuacagugcugccuugucgcauggggaucaagcagcauuguacagggcuaugaaggc   -3'    exp
    ..(((.((((((.((.(((((((((((.(((((.(...).)).))))))))))))))).))))))..)).)))    reads      mm         sample
    ........ggcuucucuacagugcugccuugu........................................    1          0          seq
    ............................................aagcagcauuguacagggcuaug.....    1          0          seq
    ............................................aagcagcauuguacagggcuauga....    12         0          seq
    .............................................agcagcauuguacaggg..........    2          0          seq
    .............................................agcagcauuguacagggc.........    26         0          seq
    .............................................agcagcauuguacUgggcu........    1          1          seq
    .............................................agcagcauuguacagggcu........    455        0          seq
    .............................................agcagcauuguacagggcua.......    393        0          seq
    .............................................agcagcauuguacagggcuau......    5178       0          seq
    .............................................agcagcauuguacagggcuaug.....    1817       0          seq
    .............................................agcagcauuguacagggcuauga....    6513       0          seq
    .............................................agcagcauuguacagggcuaugaa...    201        0          seq
    .............................................agcagcauuguacagggcuaugaaA..    18         1          seq
    ..............................................gcagcauuguacagggcuau......    5          0          seq
    ..............................................gcagcauuguacagggcuaug.....    2          0          seq
    ..............................................gcagcauuguacagggcuauga....    17         0          seq
    ...............................................cagcauuguacagggcua.......    1          0          seq
    ...............................................cagcauuguacagggcuau......    7          0          seq
    ...............................................cagcauuguacagggcuaug.....    12         0          seq
    ...............................................cagcauuguacagggcuauga....    102        0          seq
    ...............................................cagcauuguacagggcuaugaa...    6          0          seq
    ...............................................cagcauuguacagggcuaugaaA..    1          1          seq
    ................................................agcauuguacagggcuauga....    2          0          seq
```
